# Robust increase of leaf size by *Arabidopsis thaliana GRF3*-like transcription factors under different growth conditions

**DOI:** 10.1038/s41598-018-29859-9

**Published:** 2018-09-07

**Authors:** Matías Beltramino, María Florencia Ercoli, Juan Manuel Debernardi, Camila Goldy, Arantxa M. L. Rojas, Florencia Nota, María Elena Alvarez, Liesbeth Vercruyssen, Dirk Inzé, Javier F. Palatnik, Ramiro E. Rodriguez

**Affiliations:** 1 IBR (Instituto de Biología Molecular y Celular de Rosario), CONICET and Universidad Nacional de Rosario, Rosario, Argentina; 2 CONICET, Universidad Nacional de Córdoba, Centro de Investigaciones en Química Biológica de Córdoba (CIQUIBIC), Córdoba, Argentina; 3 Universidad Nacional de Córdoba, Facultad de Ciencias Químicas, Departamento de Química Biológica Ranwel Caputto, Córdoba, Argentina; 4 VIB-UGent Center for Plant Systems Biology, VIB, 9052 Ghent, Belgium; 5 Department of Plant Biotechnology and Bioinformatics, Ghent University, 9052 Ghent, Belgium; 6 Centro de Estudios Interdisciplinarios, Universidad Nacional de Rosario, Rosario, Argentina

## Abstract

An increase in crop yield is essential to reassure food security to meet the accelerating global demand. Several genetic modifications can increase organ size, which in turn might boost crop yield. Still, only in a few cases their performance has been evaluated under stress conditions. MicroRNA miR396 repress the expression of *GROWTH-REGULATING FACTOR* (*GRF*) genes that codes for transcription factors that promote organ growth. Here, we show that both *Arabidopsis thaliana At-GRF2* and *At-GRF3* genes resistant to miR396 activity (*rGRF2* and *rGRF3*) increased organ size, but only *rGRF3* can produce this effect without causing morphological defects. Furthermore, introduction of *At-rGRF3* in *Brassica oleracea* can increase organ size, and when *At-rGRF3* homologs from soybean and rice are introduced in Arabidopsis, leaf size is also increased. This suggests that regulation of *GRF3* activity by miR396 is important for organ growth in a broad range of species. Plants harboring *rGRF3* have larger leaves also under drought stress, a condition that stimulates miR396 accumulation. These plants also showed an increase in the resistance to virulent bacteria, suggesting that the size increment promoted by *rGRF3* occurs without an obvious cost on plant defenses. Our findings indicate that *rGRF3* can increase plant organ size under both normal and stress conditions and is a valuable tool for biotechnological applications.

## Introduction

The growth of plant organs is tightly controlled by their developmental program and the interaction with the environment. Leaves initiate as rod-like structures protruding from the shoot apical meristem, pass through different developmental stages and become a flat organ specialized in photosynthesis^1,2^. Multiple regulatory gene networks are known to participate in the morphogenesis of a leaf, although their precise role and interactions are unknown in many cases.

Crop yield is a highly complex trait influenced by both external and internal factors. Intrinsic Yield Genes (IYG) have been defined as those genes that produce larger organs, such as leaves, roots or seeds, when mutated or ectopically expressed^3^. In this sense, the precise modification of IYG might increase crop yield and therefore they constitute a potential source of biotechnological applications. The *GROWTH-REGULATING FACTORs* (*GRFs*) genes code for a family of plant-specific transcription factors characterized by the presence of the WRC and QLQ protein domains, which have been involved in DNA-binding and protein-protein interaction, respectively^4–9^. In *Arabidopsis thaliana*, there are nine *GRF* coding genes (*GRF1*-*9*). Seven out of them harbor a target site for microRNA miR396. At early stages of leaf development, miR396 is expressed in the distal part of the leaf, restricting the expression of the *GRFs* to the proximal part, which is coincidental with the proliferative region of the organ^10–13^. At later stages of leaf development and after the stop of cell proliferation, miR396 is expressed throughout the organ repressing *GRF* expression in maturing organs^10–12^.

The miR396-*GRF* module is present in a broad range of plants including angiosperms and gymnosperms^11,14,15^. In certain cases, the ectopic expression of the GRFs is sufficient to increase leaf size. In Arabidopsis, overexpression from the 35S promoter of *At-GRF5*^16,17^, *Brassica napus Bn-GRF2*^18^ and *Brassica rapa Br-GRF8*^19^ promotes a moderate increase of leaf size.

Modified *GRFs* have been generated with synonymous mutations in the miRNA target site to avoid the post-transcriptional repression mediated by miR396^12,20^. The observation that plants harboring these miR396-resistant versions of *At-rGRF2* (*rGRF2*) or *At-rGRF3* (*rGRF3*) have larger leaves with respect to wild type plants, indicates that miR396 normally restricts organ size through the repression of the genes coding for GRF transcription factors. In good agreement with these results, plants overexpressing miR396^12,21^ or single *grf5*^17^ and multiple *grf1 grf2 grf3*^5^ knock outs have smaller organs.

However, increased levels of the GRFs not always results in larger organs in Arabidopsis, as overexpression of *Oryza sativa Os-GRF1* caused pleiotropic defects, including curled leaves, delayed flowering and defects in carpel development^4^. Results in crops have also been variable. Overexpression of *Zm-rGRF1* increased maize leaf size, while it also caused additional detrimental phenotypes such as large macrohairs covering the glumes and the ear rachis that reduced fertility^15^, while overexpression of *Zm-GRF10*, which lacks a transactivation domain, reduced maize leaf size^22^. Furthermore, high levels of At-GRF7 and At-GRF9 caused no major increase of Arabidopsis leaf size^23–25^. Interestingly, At-GRF7 has been implicated in the response of plants to osmotic stress^23^, while At-GRF9 has been claimed to be a growth repressor^26^. Furthermore, in certain organs and conditions, the GRFs can affect both cell number and size^27,28^.

The capacity of certain GRFs to increase leaf size *per se* suggests that they can act as IYG increasing plant organ size, and therefore they could be a valuable tool for biotechnological applications. Still, not all the GRFs have a positive impact on organ size, and some of them have even deleterious effects. Here, we characterized different members of the *GRF* family in Arabidopsis and found that the *At-GRF3* gene decoupled from miR396 regulation consistently increase organ size in *Arabidopsis thaliana*, an ability that likely depends on the protein sequence of the transcription factor. We also show that a miR396-resistant *GRF3* can increase leaf size, root length and seed size in transgenic *Brassica oleracea*. Furthermore, we found that plants expressing the *rGRF3* transgene still have an increase in leaf size under mild drought stress and show enhanced resistance to certain plant pathogens. We conclude that GRF transcription factors similar to *At-GRF3* can be used to increase plant organ size in *Brassicaceae* species without an obvious deleterious impact in plant fitness.

## Results

### Broad distribution of GRF regulation by miR396 in angiosperms

**Figure 1:**
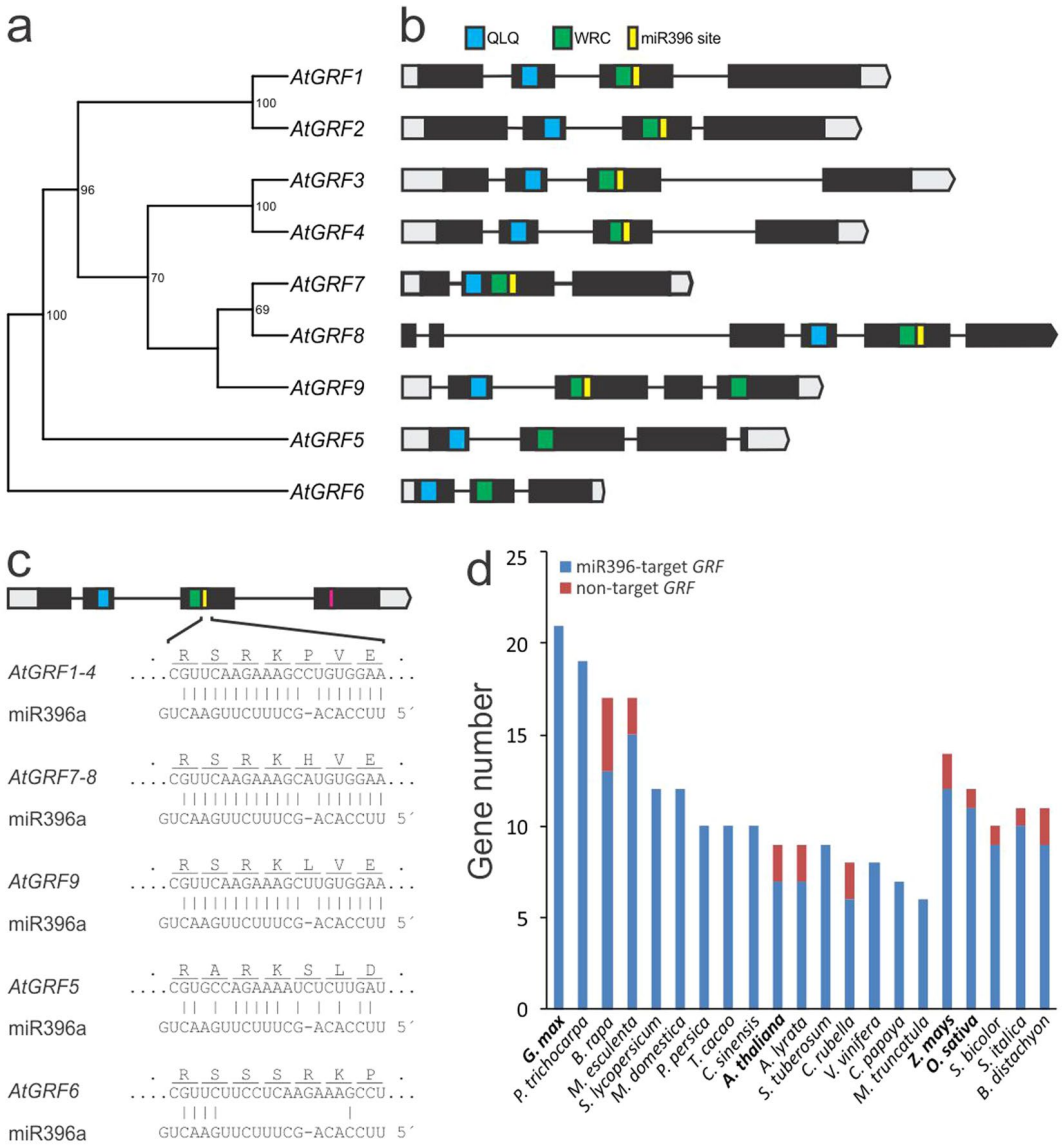
Broad control of GRF transcrition factors by miRNA miR396. (**a**) Phylogenetic tree using the full-length amino acid sequences of the Arabidopsis GRFs constructed by the neighbor joining method. Bootstrap support greater than 50% are indicated on nodes. (**b**) Scheme representing the exon-intron structure, the localization of the WRC and QLQ protein domains and the miR396-binding site. (**c**) Scheme representing a typical *GRF* gene and a detailed view of the interaction of Arabidopsis GRFs with miR396. (**d**) Distribution of GRFs in representative plants species and the occurrence of miR396 regulation among them.

There are nine *GRFs* in Arabidopsis (Fig. 1a,b), and seven of them have a target site for miR396 located in the region that codes for the carboxyl end of the WRC domain (Fig. 1b,c). The miRNA target site is identical in the different *GRFs* with an exception at position eight where there is a C in *GRFs* 1-4, an A in *GRFs 7*-*8*, and a U in *GRF9* (Fig. 1c). This variable base is located in a bulged position with respect to the miRNA so that the interaction with the miRNA is quite similar for all the transcription factors, however, the encoded amino acid sequence in the carboxyl terminal side of WRC domain varied (Fig. 1c).

Interestingly, the Arabidopsis *GRF* gene structures revealed differences in the exon-intron organization and in the distribution of the protein domains that define the family (Fig. 1b). We analyzed the occurrence of the miRNA target site in *GRFs* of different angiosperms (Fig. 1d and Supplementary Table S1). In many species, such as *Populus trichocarpa* (poplar), *Glycine max* (soybean) and *Medicago truncatula*, all the *GRFs* have a miR396-binding site (Fig. 1d), while in others like Arabidopsis and rice a few genes lack this sequence. Interestingly, there is a sequence remotely resembling a miRNA target site in Arabidopsis *GRF5*, one of the two *GRFs* that lack of miR396 regulation in this species (Fig. 1c). These findings indicate a broad distribution of *GRF* regulation by miR396 and suggest that this might be the default state of these transcription factors in angiosperms.

### Differential GRF activity among family members

**Figure 2:**
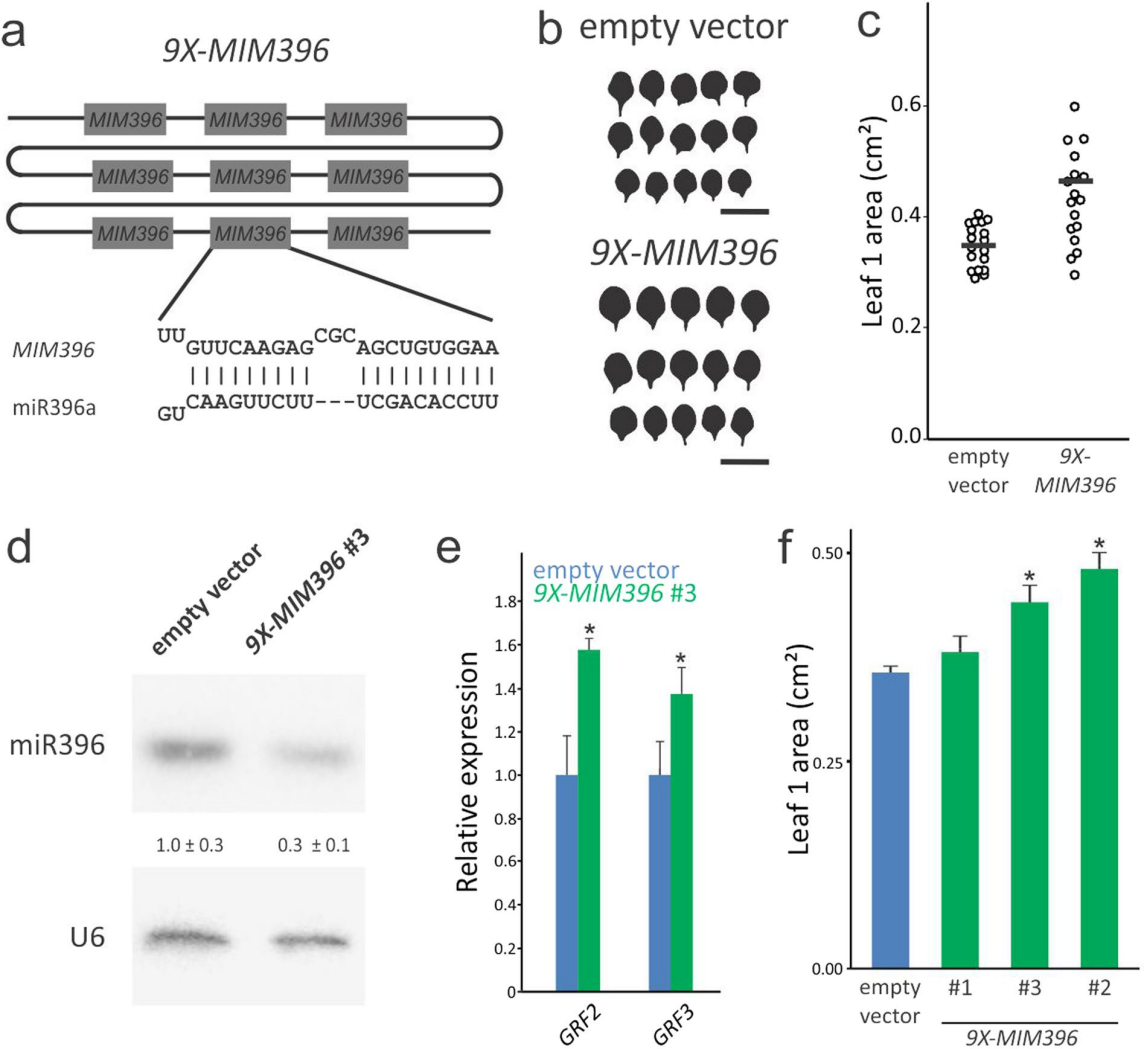
miR396 limits leaf size in Arabidopsis. (**a**) Scheme of the multi-miR396 target mimic (*9X-MIM396*) prepared against miR396. (**b**,**c**) Fully expanded leaf 1 size distribution in a population of Arabidopsis primary transgenic plants transformed with the empty vector or with the *9X-MIM396* construct. In panel b, each silhouette of leaf 1 belongs to an independent primary transgenic plant transformed with the indicated vector. In the scatterplots in panel c, each circle indicates the size of a single leaf, while the horizontal solid bar represents the sample median. Bar = 1 cm. (**d**) Expression levels of miR396 in control plants transformed with the empty vector and *9X-MIM396* #3 plants. The miR396 levels were estimated by small RNA blots and the abundance relative to control plants is indicated by numerals. The data shown are mean ± SEM of three biological replicates. A probe against U6 snRNA was used as a loading and blotting control. The blot shown in the figure is a representative pair of samples from control and *9X-MIM396* #3 plants. (**e**) Expression levels of *GRF2* and *GRF3* in control plants transformed with the empty vector and *9X-MIM396* #3 plants. The *GRF* levels were estimated by RT-qPCR and normalized to control plants. The data shown are mean ± SEM of three biological replicates. Asterisks indicate significant differences from the control plants as determined by Student’s t-test (*P* < 0.05). (**f**) Leaf 1 size in control (empty vector) and three independent T3 homozygous transgenic lines transformed with *9X-MIM396*.

To study the loss of function of *MIR396*, we prepared and characterized plants expressing a miR396 target mimic (MIM396) that consists of a noncoding RNA that binds to the miRNA and blocks its function and/or promotes its degradation^29,30^. We prepared a MIM396 harboring 9 sites that can act as a sponge for miR396 (*9X-MIM396*) (Fig. 2a). First, we analyzed *ca*. 20 primary transgenic plants transformed with the *9X-MIM396*. We observed that this construct consistently caused an increase in leaf area that reached 70% in the most extreme cases (Fig. 2b,c). The area of the first leaf was increased when independent homozygous transgenic lines were analyzed (Fig. 2f). Further molecular analysis of *9X-MIM396* #3 line revealed a reduction of 70% in miR396 expression (Fig. 2d) and a 40% increase of *GRF2* and *GRF3* transcript levels (Fig. 2e), in agreement with the observed phenotypes (Fig. 2b,c and f).

Previous results have shown that the ectopic expression of *GRF* caused different effects, e.g., while in Arabidopsis *rGRF2* or *rGRF3* expressed from their own promoter or *35S:GRF5* caused an increase of leaf size^12,16,20^, overexpression of *GRF7* and *GRF9* did not^23–25^. Furthermore, overexpression of certain *GRFs* from other species might also inhibit growth^4^. The combined observation that most of the *GRFs* are regulated by miR396 in angiosperms and that the developmental phenotype of *9X-MIM396* was an increase of leaf size, showed that the miR396-*GRF* module controls organ size *in vivo*. These results prompted us to focus on *rGRF2* and *rGRF3* in more detail, especially in the light of the potential biotechnological application to increase the size of leaves and other organs.

**Figure 3:**
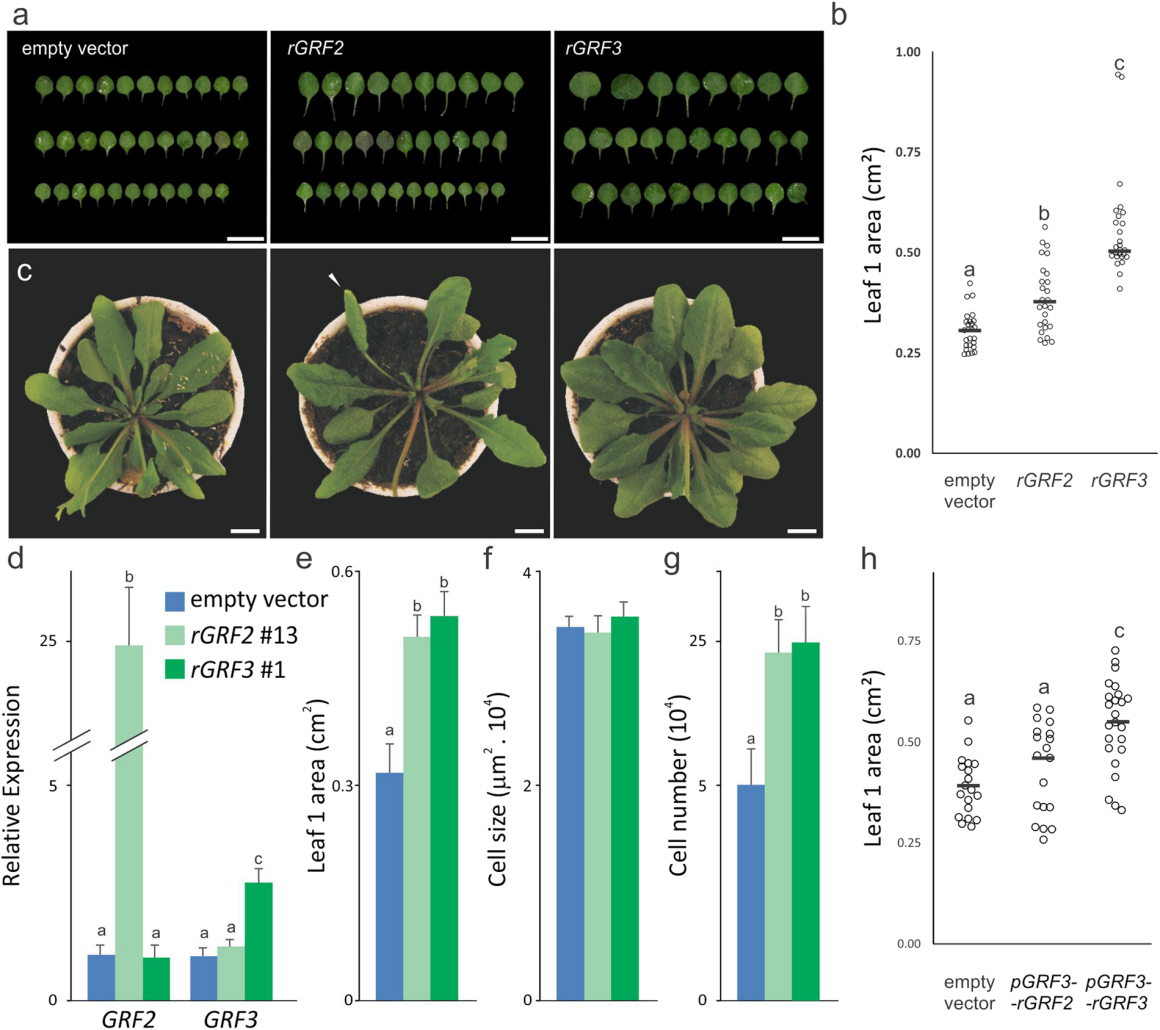
Superior capacity of *rGRF3* compared to *rGRF2* in increasing leaf size. (**a**,**b**) Fully expanded leaf 1 size distribution in a population of Arabidopsis primary transgenic plants transformed with the empty vector, *rGRF2* or *rGRF3*. In the scatterplots in panel b, each circle indicates the size of a single leaf from an independent T1 transgenic plant, while the horizontal solid bar represents the sample median. Different letters indicate significant differences, as determined by ANOVA followed by Tukey’s multiple comparison test (P < 0.05). Bars = 1 cm. (**c**) 30-days old plants transformed with the empty vector, *rGRF2* or *rGRF3*. Note the leaf shape changes induced by *rGRF2* only (arrowhead), including long and twisted petioles with downward curled leaves. Bars = 1 cm. (**d**) Expression levels of At-*GRF2* and At-*GRF3* in homozygous T3 transgenic plants transformed with the empty vector or *rGRF2* (rGRF2 #13) or rGRF3 (rGRF3 #1). The *GRF* levels were estimated by RT-qPCR and normalized to control plants (empty vector). The data shown are mean ± SEM of three biological replicates. Different letters indicate significant differences as determined by ANOVA followed by Tukey’s multiple comparison test (P < 0.05). (**e**–**g**) Leaf 1 size (**e**), palisade cell size (**f**) and estimated palisade cell number (**g**) in selected *rGRF2 #13* and *rGRF3* #1plants. Different letters indicate significant differences as determined by ANOVA followed by Tukey’s multiple comparison test (P < 0.05). (**h**) Fully expanded leaf 1 size distribution in a population of primary transgenic Arabidopsis plants transformed with *rGRF2* or *rGRF3* under the *GRF3* promoter. In the scatterplots each circle indicates the size of a single leaf from an independent T1 transgenic plant, while the horizontal solid bar represents the sample median. Different letters indicate significant differences, as determined by ANOVA followed by Tukey’s multiple comparison test (P < 0.05). Bars = 1 cm.

First, we analyzed approximately 30 primary transgenic plants harboring *rGRF2* or *rGRF3* (Fig. 3a,b) expressed from their own promoters. Although both constructs were able to increase leaf area, the effect caused by *rGRF3* was significantly higher than the effect obtained with *rGRF2*. We then selected *rGRF2* and *rGRF3* homozygous T3 transgenic lines and determined the *GRFs* transcript levels (Fig. 3d and Supplementary Fig. 1). We found that a 2-fold increase in *GRF3* expression was sufficient to change leaf area in 70%, while an increase of more than 25-fold in *GRF2* transcripts is required to cause a similar increase in organ size (Fig. 3d,e). In both cases, the increase in organ size was due to a similar raise in the number of cells, with no appreciable change in cell size (Fig. 3e,f and g).

Further analysis of *rGRF2* plants revealed that the leaves had morphological defects, including long and twisted petioles with downward curled leaves (Fig. 3c). On the other hand, no obvious morphological changes were observed in *rGRF3* plants besides the increase in leaf area. We considered that the high levels of *GRF2* required to increase leaf size, caused also additional morphological defects in plant development. To confirm that the promoter was not the cause of the observed differences between *rGRF2* and *rGRF3*, we expressed both *rGRF2* and *rGRF3* from the *GRF3* promoter and observed that *pGRF3:rGRF3* caused a larger increase in leaf area than *pGRF3:rGRF2* (Fig. 3h). Therefore, both expression levels and GRF protein sequences should be considered to efficiently enhance plant organ size. As shown here, although both *rGRFs* can be used to increase organ size, *rGRF3* is a more active and specific enhancer of plant organ size.

### Robust increase in leaf size by expression of Arabidopsis *GRF3*-like sequences decoupled of miR396 regulation

**Figure 4:**
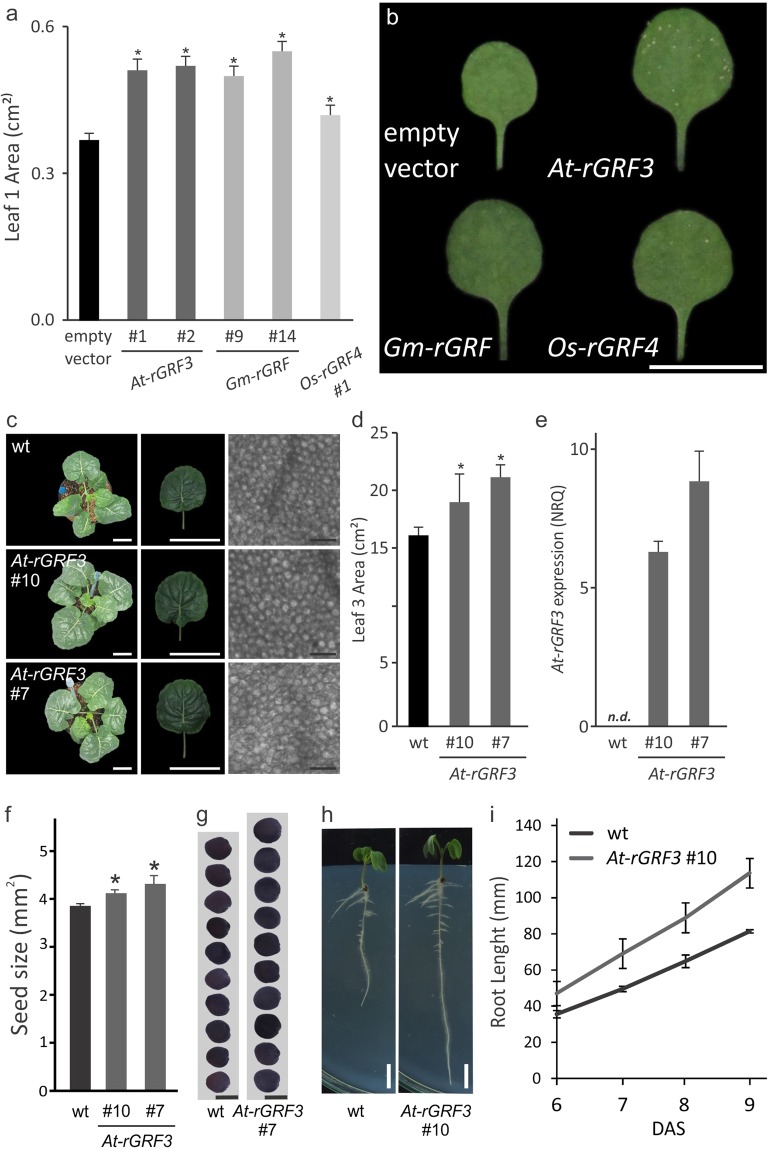
*rGRF3*-like genes increase organ size in heterologous species. (**a**) Area of fully expanded leaf 1 of transgenic Arabidopsis plants expressing *At-rGRF3* or selected orthologues from soybean (*Gm-rGRF*) or rice (*Os-rGRF4*). The data shown are mean ± SEM of 20 biological replicates. Asterisks indicate significant differences from plants transformed with the empty vector as determined by Student’s t-test (*p* < 0.05). (**b**) Leaf 1 of transgenic plants expressing *At-rGRF3* or the corresponding orthologues from soybean (*Gm-rGRF*) or rice (*Os-rGRF4*). Bar = 1 cm. (**c**) Wild-type and transgenic *Brassica oleracea* plants expressing *At-rGRF3*. To the left, 4 week-old whole plants, in the middle, fully expanded leaf 3 and to the right, a paredermal view of palisade cells from fully expanded leaf 3. White bars = 5 cm; Black bars = 0.05 mm. (**d**) Size of leaf 3 of 4 week-old wt and *rGRF3* transgenic *B. oleracea* plants. The data shown are mean ± SEM of 10 biological replicates. Asterisks indicate significant differences from the wt as determined by Student’s t-test (P < 0.05). (**e**) Expression of *rGRF3* in *B. oleracea* transgenic plants as estimated by RT-qPCR. Data are mean ± SEM of 3 biological replicates. (**f**,**g**) Seed size of wt and *rGRF3* #10 and #7 *B. oleracea* plants. The data shown in f are mean ± SEM of 30 biological replicates. Asterisks indicate significant differences from the wt control plants as determined by Student’s t-test (P < 0.05). Bar = 2 mm. (**h**,**i**) Root architecture (**h**) and root growth (**i**) of *B. oleracea* plants expressing *rGRF3*. The data shown are mean ± SEM of 6 biological replicates. Asterisks indicate significant differences from wt control plants as determined by Student’s t-test (P < 0.05). Bars = 1 cm.

Given the potential use of *rGRF3* as a tool to promote plant organ size, we decided to express Arabidopsis *GRF3*-like sequences from selected crops in Arabidopsis. We analyzed the databases for *GRF* transcription factor sequences from rice^6^ and soybean^8^ and selected those with the highest similarities to *At-GRF3* (Supplementary Table S2). Then, we expressed soybean and rice Arabidopsis *GRF3*-like coding sequences in Arabidopsis under the *At-GRF3* promoter. As both genes have a miR396 target site, we introduced synonymous mutations to avoid the recognition by the small RNA. We observed an increase in leaf size caused by both soybean and rice *rGRF3*-like transcription factors (Fig. 4a,b).

To evaluate the effect of *rGRF3* on leaf size in another species, we expressed the transcription factor in *Brassica oleracea* transgenic plants (Fig. 4e). Analysis of two independent transgenic lines revealed a significant increase in leaf area of 20 and 32% (Fig. 4c,d). Further characterization of the transgenic leaves revealed that the increase in leaf size was caused by a higher number of cells and not by an effect in cell size (Fig. 4c). The evaluation of root growth in *B. oleracea rGRF3* #10 plants showed that the primary root of these plants elongated at a higher rate than control plants (Fig. 4h,i). Furthermore, these plants also had an increase of 10% in seed size (Fig. 4f,g). Taken together, these results show that *AtGRF3*-like sequences from various species when decoupled from miR396 regulation robustly increase the size of several organs, including leaves, roots and seeds.

### Role of the *miR396-GRF* node under stress conditions

**Figure 5:**
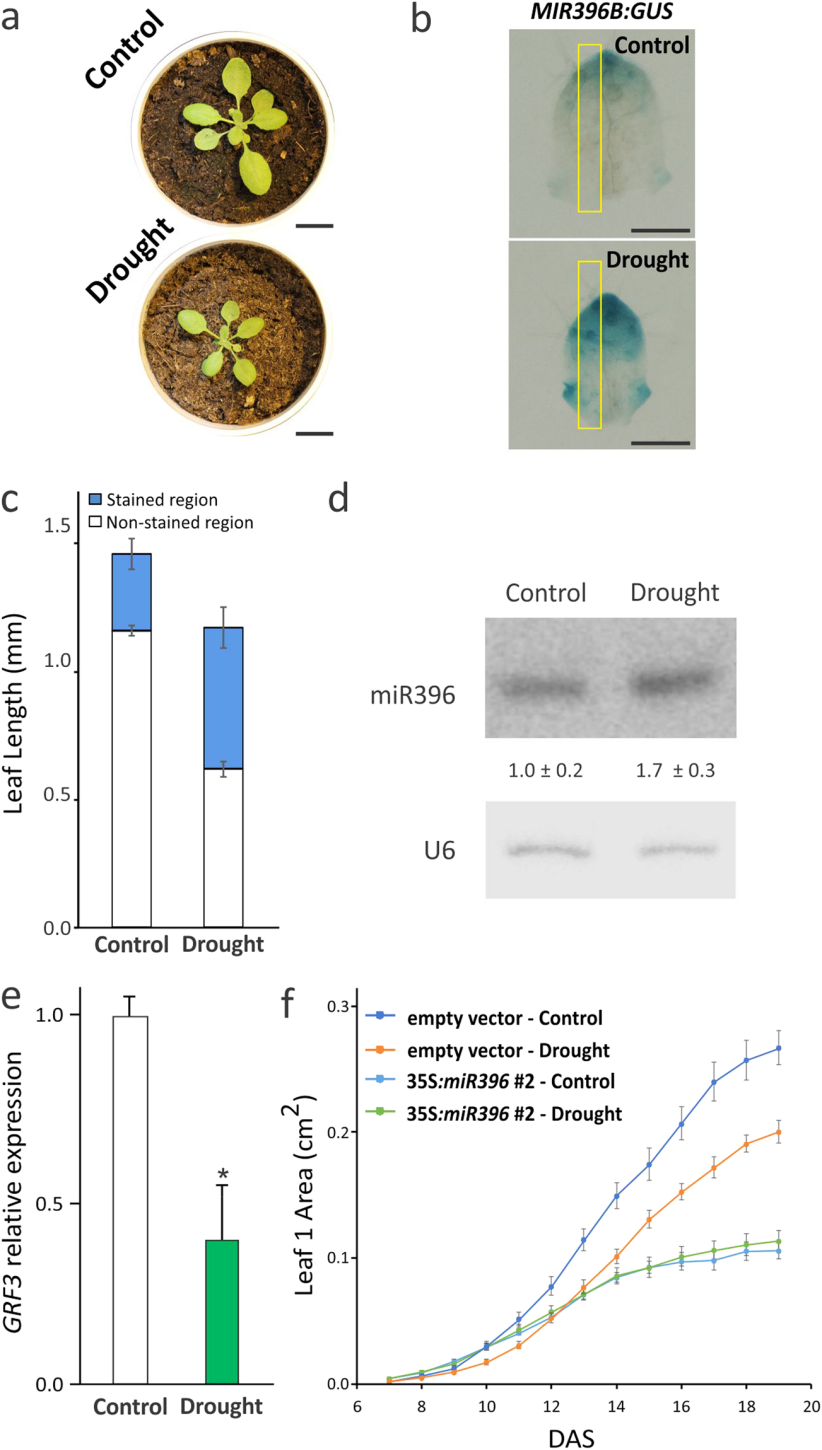
Response of the *miR396-GRF* system to drought stress. (**a**) Decrease in rosette area after a mild drought stress in 19-day-olds control plants transformed with the empty vector. Bar = 1 cm. (**b**) Premature induction of the *MIR396B* gene during a mild drought stress. A *MIR396B:GUS* reporter was used to monitor miR396b expression under normal and drought conditions. Staining was performed in 11 days old plants. Bars = 0.5 mm. (**c**) Size of the GUS-stained (blue) and non-stained regions (white) measured along the length of the leaves under normal and drought conditions. GUS staining was measured in stained leaves as those of panel b in a defined area along the leaf length. This area is depicted by a yellow box in panel b. The data shown are mean ± SEM of 20 leaves. (**d**) MiR396 induction after mild drought treatment in developing leaves. miR396 accumulation in total RNA extracted from leaves as those of panel b was estimated by small RNA blots. The abundance relative to control plants is indicated by numerals. The data shown are mean ± SEM of two biological replicates. A probe against U6 snRNA was used as a loading and blotting control. The blot shown in the figure is a representative pair of samples from plants grown in control or drought conditions. (**e**) Repression of *At-GRF3* expression in plants grown under mild drought stress. The chart indicates the *At-GRF3* expression levels in plants grown under control and mild drought conditions. The data shown, normalized to the expression value under control conditions, are mean ± SEM of 3 biological replicates. Asterisks indicate significant differences from control plants as determined by Student’s t- test (P < 0.05). (**f**) Dynamics of leaf 1 growth in control and drought conditions of control (empty vector) or 35S:*miR396* #2 plants. Leaf size was monitored from 7 to 19 days after sowing under control and mild drought in the WIWAM platform. The data shown are mean ± SEM of 10 biological replicates.

Drought stress is a fairly complex situation triggering different response pathways according to the magnitude of the stress and the developmental stage of the plant^31^. In particular, water limiting conditions repress cell proliferation and expansion in developing organs and/or induce a complex array of tolerance and survival responses in mature organs^31–33^. Under field conditions limited water availability usually reduces plant growth, biomass accumulation and, therefore, seed yield^34^. We evaluated the response of plants to moderated drought stress consisting of a 55% reduction in soil water content using the automated phenotyping platform WIWAM^34^. Under these conditions, control plants transformed with the empty vector had a reduction in rosette area of 40% (Fig. 5a) and a reduction of 35% in leaf 1 area (Fig. 5f).

We looked at the expression of *MIR396B* using the transcriptional reporter *MIR396B:GUS* that allowed us to monitor the expression of the most abundant miR396-coding gene in leaves. At 11 days after sowing (DAS) in well wattered pots *MIR396B* is expressed at low levels in the proximal end of leaf 3, that is still in a proliferative state (Fig. 5b). The highest expression of *MIR396B:GUS* is found in the distal zone of the leaf in which cells exited the cell cycle. When plants were subjected to mild drought stress leaf size was reduced and *MIR396B:GUS* expression was detected in a larger distal leaf area with a more intense staining (Fig. 5b,c). Accordingly, small RNA blots revealed an increase of 70% in the mature miR396 levels in developing leaves from stressed plants (Fig. 5d). In contrast, *At-GRF3* transcript levels decrease by approximately 60% (Fig. 5e). Altogehter, these results suggest that the miR396 network responds to drougth stress. We then evaluated the role of the miR396-*GRF3* regulatory node during drought stress. To do this, we first analyzed the response to mild drought stress of 35S:*miR396* plants, which have small leaves due to the overexpression of the miRNA and the repression of the *GRFs*^12^ (Fig. 5f). When drougth stress is imposed to these plants, no further reduction in organ size could be observed (Fig. 5f), indicating that transgenic miR396 overexpression masks the effects derived from the endogenous induced *MIR396B*.

**Figure 6:**
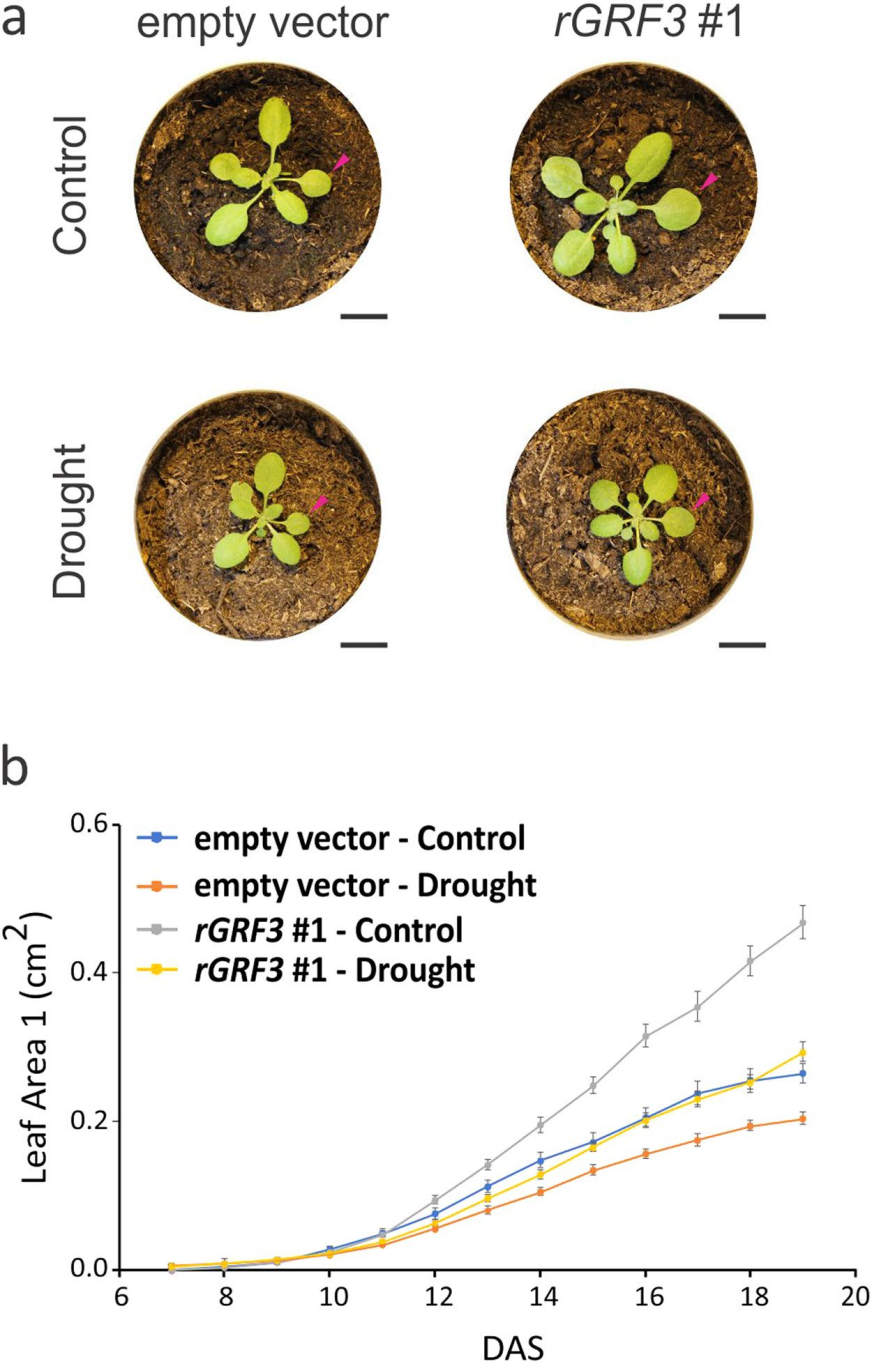
*rGRF3* ameliorates the effect of drought on leaf size. (**a**) 19 DAS rosettes of control (empty vector) and *rGRF3* plants grown under control and drought conditions. Arrowheads indicate leaf 1. Bars = 1 cm. (**b**) Dynamics of leaf 1 growth from control and *rGRF3* plants grown in control and drought conditions. Leaf size was monitored from 7 to 19 days after sowing under control (**b**) and mild drought (**c**) in the WIWAM platform. The data shown are mean ± SEM of 10 biological replicates.

When the stress was applied to *rGRF3* lines (Fig. 6), leaf size was reduced with respect to *rGRF3* plants grown under control conditions. However, at the end of the experiment, 19 days after sowing, *rGRF3* leaves were significantly larger than leaves from the control plants growing under mild drought (Fig. 6a,b). Notably, we did not find differences in the plastochron length caused by higher GRF3 expression levels or the mild drought conditions assayed. Therefore, the differences in rosette size observed are mainly due to changes in leaf organ size rather than in changes in rosette architechture (Supplementary Fig. 2).

Having tested mild drought as an abiotic stress, we turned to pathogen infections to evaluate the susceptibility of *rGRF3* plants to biotic stress. This is of particular interest, as diverting energy to organ growth might compromise plant defenses to pathogen infections. To do this, adult plants were infiltrated with the hemibiotrophic virulent bacteria *Pseudomonas syringae* pv. tomato DC3000 (Pst) and pathogen content was quantified at 2, 3 and 5 days post infection (dpi). Interestingly, Pst titers were lower in *rGRF3* than in wild type plants, and these differences were maintained also at late stages of infection (9 times difference at 5 dpi). This indicated a positive effect of GRF3 on the immune pathways responsible to counteract pathogen proliferation (Supplementary Fig. 3). Altogether, these data indicate that *rGRF3* can be a valuable tool that might ameliorate the effects of drought stress on organ growth and increase leaf size under both normal and stressfull conditions.

## Discussion

Here, we show that *At-GRF3*-like sequences from various species like soybean and rice can systematically increase leaf, seed and root size in *Brassicaceae* species, i.e. *Arabidopsis thaliana* and *Brassica oleracea*, when decoupled from miR396 regulation.

Previous results using GRFs to boost plant growth and yield have generated inconsistent results. On the one hand, *MIM396* mimics increased tomato organ size^35^, and natural occurring alleles of *Os-GRF4*, a miR396-targeted *GRF* from rice with homology to *At-GRF3*, increases leaf size, grain weight and rice yield^27,28,36,37^. On the other hand, overexpression of *Os-GRF1* in Arabidopsis^4^ or *Zm-GRF1* in maize^15^ lead to developmental defects that affected plant fertility and seed production. Our results suggest that it is relevant to choose the proper *GRF* coding sequence for genetic engineering in the context of an adequate expression system in order to increase plant organ size and eventually crop yield. Also, they suggest that the controlled expression of *At-GRF3*-like genes have the highest chance to boost organ growth with no associated developmental defects.

Crop plants under field conditions often face drought stress that reduce plant growth and yield. An integral part of drought responses in plants is growth repression to limit shoot size, reduce evaporation surface and match sink-tissue demands to the scarce resources available due to stomatal and metabolic restrictions^31^. This occurs due to premature transitions from cell proliferation to cell expansion combined with a reduced magnitude of cell expansion^33,38^.

In leaves from dicot and monocot species, miR396 is expressed in low levels in meristems and induced at later developmental stages to promote the transition from cell proliferation to cell elongation and maturation^11,12,39,40^. We found that miR396 was prematurely induced while its target *At-GRF3* was repressed under mild drought. These results, together with the finding that plants expressing a miR396-resistant *At-GRF3* were more tolerant to the stress, indicate that miR396 might be part of the genetic mechanisms that limit leaf size during water limitation.

Microarray and RNA-seq studies have shown that MIR396 can respond to other abiotic stresses, such as aluminum^41^, cadmium^42^, salt^43^ and alkali stress^44^ (Ding *et al*., 2009; Gao *et al*., 2010). It has been recently shown, both in Arabidopsis and maize, that miR396 levels are prematurely induced by UV-B in developing leaves. This induction causes the repression of the *GRFs* expression and a decrease of cell proliferation, leading to smaller leaves with fewer cells^45,46^.

Plant expressing *GRF3* decoupled of miRNA regulation reduced their size upon mild drought stress treatment. Still, *rGRF3* leaves were significantly larger than those from the control plants grown under drought stress. Therefore, *At-rGRF3* is able to increase plant organ size under both optimal and adverse conditions.

A particular role has been established for At-GRF7 in Arabidopsis in the context of stress responses. Mutants in this gene have higher expression levels of stress-responsive genes in developing organs, including *DREB2A*, a master regulator of water limitation responses. At- GRF7 has been shown to act as a transcriptional repressor of *DREB2A* and other stress-responsive genes to avoid the detrimental effects of stress responses on cell proliferation and organ growth^23^.

Recently, it has been shown that *MIM396* plants, with low miR396 activity, develop fast and strong immune responses and increased resistance to nectrotrophic and hemibiotrophic fungi, apparently by triggering defense priming^47^. Our results suggest that *At-GRF3* could mediate similar responses as well. *rGRF3* plants, in which miR396-mediated repression of *GRF3* is avoided, presented increased resistance to virulent *Pst* suggesting a positive effect of this transcription factor on defenses against hemibiotrophs. Interestingly, our results indicate that *rGFR3* improves immune responses at the same time that it boosts plant growth. This contrasts with many examples illustrating the costs of increasing defenses, where activation of immune pathways under non-stress conditions produces a fruitless diversion of energy that causes a negative effect on plant growth and fitness^48^. In turn, other plants with enhanced resistance and no fitness cost have also been described, including *MIM396* itself^47^ and overexpressors of *NPR1*^49^. Then, it is expected that *rGRF3* plants may achieve a suitable balance to improve both stress response and growth under adverse conditions, however, we cannot exclude that *GRF3* has different roles in these processes.

In summary, our results indicate that it might be possible to increase plant size through the genetic engineering of Arabidopsis *GRF3*-like transcription factors, even under stress conditions. It’s worth to mention, that the gene manipulations required are limited to only a few base pair substitutions in the properly chosen *GRF*, making possible to use modern gene editing technologies for the fast introduction of these traits in crop species with desired characteristics.

## Methods

### Plant materials and growth conditions

Arabidopsis of the Col-0 accession was used in all the experiments. See Supplementary Table S4 for a description of the plasmids used to generate the transgenic lines characterized in this study. *Brassica olaracea* cultivation and transformation was performed as described in (Moloney *et al*., 1989). For leaf analysis plants were grown on soil while for root analysis, surface-sterilized seeds were sown on solid medium containing 1X Murashige and Skoog salt mixture, 1% sucrose, 2.3 mM 2-(Nmorpholino) ethanesulfonic acid (pH 5.8) and 1% agar. In all cases plants were grown at 16 h light/8 h dark photoperiod at 23 °C and a light intensity of 100 μmol quanta m^−2^ s^−1^. Leaf and seed size and root length were measured using FIJI^50^. In all cases, photographs of rosettes, leaves, seeds or roots were taken next to a ruler that was used as a reference to convert pixels to the corresponding metric unit. Later, organ size or length was measured in pixels in Fiji and, finally, these values were converted to metric units using the conversion factor obtained.

### Expression analysis

RNA was extracted using TRIzol reagent (Invitrogen) and 0.5–1.0 μg of total RNA was treated with RQ1 RNase-free DNase (Promega, http://www.promega.com). Next, first-strand cDNA synthesis was carried out using SuperScript III Reverse Transcriptase (Invitrogen) with the appropriate primers. PCR reactions were performed in a Mastercycler ep realplex thermal cycler (Eppendorf, http://www.eppendorf.com) using SYBRGreen I (Roche, http://www.roche.com) to monitor double-stranded (ds)DNA synthesis. Quantitative (q)PCR of each gene was carried out in at least three biological replicates, with technical duplicates for each biological replicate. After normalizing using *PP2A* (*PROTEIN PHOSPHATASE 2A*) for Arabidopsis^51^ or *UBC* (*UBIQUITIN CONJUGATING ENZYME)* for *Brassica oleracea*^52^ transcript levels relative to the selected control genotype or condition was determined for each sample. Primer sequences are given in Supplementary Table S3.

MiR396 accumulation was estimated by small RNA blots performed as described previously^12^. Briefly, 5 μg of total RNA was resolved on 17% polyacrylamide denaturing gels containing 7 M urea. Blots were hybridized using a radioactively labelled locked nucleic acid (LNA, Exiqon, Denmark) oligonucleotide probe designed against miR396 or a probe against U6 snRNA as a loading and blotting control (5′-CTCGATTTATGCGTGTCATCCTTGC). Phosphorimaging were obtained with a Typhoon (GE) and the signal intensity of ach band were determined using the GelQuant. NET software (biochemlabsolutions.com). The values of the bands detected with the miR396 probe were first normalized to the signal obtained with the U6 snRNA probe and then relativized to the selected control genotype or condition.

To visualize GUS reporter activity, transgenic plants were subjected to GUS staining and the staining intensity along the length of the leaf was estimated as described previously^53^.

### Microscopy techniques

To obtain paradermal views of palisade cells, leaves were fixed with formalin-acetic acid-alcohol and cleared with chloral hydrate solution (200 g chloral hydrate, 20 g glycerol, and 50 ml H2O) as described^17^. Palisade leaf cells were observed using differential interference contrast microscopy in a Olympus BH2 microscope. The density of palisade cells per unit area was determined, and the area of the leaf blade was divided by this value to calculate the total number of palisade cells in the sub epidermal layer. To determine the cell area, 20 palisade cells were measured in each leaf.

### Stress Treatments

Plants were germinated on soil-filled 96-well plates after stratification at 4 °C in the dark. Seedlings were transferred to pots at 4 days after sowing (DAS). Before transfer, the relative water content of the pots was set at 1.19 g water g^−1^ dry soil for the mild drought treatment while the control condition was set at 2.19 g water g^−1^ dry soil. Once the seedlings were transferred to these pots they were placed on the automated phenotyping platform WIWAM^34^. The water content of the soil was kept constant until 8 DAS, after which it was lowered daily to 1.02 g water g^−1^ dry soil for the mild-drought-treated plants. This phenotyping platform is designed to monitor vegetative development as images of the rosette of each plant are taken daily until 19 DAS. Leaf 1 area was measured from these images to monitor organ growth during the experiment. Also, the plastochron length was estimated by registering the time when each consecutive leaf reached at least 1 mm in length.

Susceptibility to *Pseudomonas syringae* pv. tomato DC3000 was determined using 4 weeks old plants. Bacterial suspensions (5.10^5^ cfu/ml) were infiltrated into adult leaves using a syringe. Pathogen growth was determined as previously described^54^, using a set of six leaf-disks (4 mm^2^) of independent plants per sample.

### Sequence analysis

Multiple sequence alignments and phylogenetic trees were obtained using the Clustal Omega^55^ and PHYLIP^56^ softwares using the neighbor joining method. Bootstrap analysis was conducted to measure node robustness using 1000 replicates.

### Data Availability

The datasets generated during and analyzed during the current study are available from the corresponding author on request.

## Electronic supplementary material

Supplementary Info

## References

[1] Donnelly PM, Bonetta D, Tsukaya H, Dengler RE, Dengler NG (1999). Cell cycling and cell enlargement in developing leaves of Arabidopsis. Developmental biology.

[2] Rodriguez RE, Debernardi JM, Palatnik JF (2014). Morphogenesis of simple leaves: regulation of leaf size and shape. Wiley interdisciplinary reviews. Developmental biology.

[3] Gonzalez N, Beemster GT, Inze D (2009). David and Goliath: what can the tiny weed Arabidopsis teach us to improve biomass production in crops?. Current opinion in plant biology.

[4] van der Knaap, E., Kim, J. H. & Kende, H. A novel gibberellin-induced gene from rice and its potential regulatory role in stem growth. Plant physiology 122, 695–704, https://www.ncbi.nlm.nih.gov/pmc/articles/PMC58904/ (2000). 10.1104/pp.122.3.695 PMC58904 10712532

[5] Kim JH, Choi D, Kende H (2003). The AtGRF family of putative transcription factors is involved in leaf and cotyledon growth in Arabidopsis. Plant J.

[6] Choi D, Kim JH, Kende H (2004). Whole genome analysis of the OsGRF gene family encoding plant-specific putative transcription activators in rice (Oryza sativa L.). Plant & cell physiology.

[7] Zhang D-F (2008). Isolation and characterization of genes encoding GRF transcription factors and GIF transcriptional coactivators in Maize (Zea mays L.). Plant Science.

[8] Zhang H (2011). PlantTFDB 2.0: update and improvement of the comprehensive plant transcription factor database. Nucleic acids research.

[9] Rodriguez RE, Schommer C, Palatnik JF (2016). Control of cell proliferation by microRNAs in plants. Current opinion in plant biology.

[10] Schommer C, Debernardi JM, Bresso EG, Rodriguez RE, Palatnik JF (2014). Repression of Cell Proliferation by miR319-Regulated TCP4. Molecular plant.

[11] Debernardi JM, Rodriguez RE, Mecchia MA, Palatnik JF (2012). Functional specialization of the plant miR396 regulatory network through distinct microRNA-target interactions. PLoS genetics.

[12] Rodriguez RE (2010). Control of cell proliferation in *Arabidopsis thaliana* by microRNA miR396. Development (Cambridge, England).

[13] Das Gupta M, Nath U (2015). Divergence in Patterns of Leaf Growth Polarity Is Associated with the Expression Divergence of miR396. The Plant cell.

[14] Jones-Rhoades MW (2012). Conservation and divergence in plant microRNAs. Plant molecular biology.

[15] Nelissen H (2015). Dynamic Changes in ANGUSTIFOLIA3 Complex Composition Reveal a Growth Regulatory Mechanism in the Maize Leaf. The Plant cell.

[16] Gonzalez N (2010). Increased leaf size: different means to an end. Plant physiology.

[17] Horiguchi G, Kim GT, Tsukaya H (2005). The transcription factor AtGRF5 and the transcription coactivator AN3 regulate cell proliferation in leaf primordia of *Arabidopsis thaliana*. Plant J.

[18] Liu J (2012). The BnGRF2 gene (GRF2-like gene from Brassica napus) enhances seed oil production through regulating cell number and plant photosynthesis. Journal of experimental botany.

[19] Wang F (2014). Genome-wide identification and analysis of the growth-regulating factor family in Chinese cabbage (Brassica rapa L. ssp. pekinensis). BMC Genomics.

[20] Debernardi JM (2014). Post-transcriptional control of GRF transcription factors by microRNA miR396 and GIF co-activator affects leaf size and longevity. Plant J.

[21] Liu D, Song Y, Chen Z, Yu D (2009). Ectopic expression of miR396 suppresses GRF target gene expression and alters leaf growth in Arabidopsis. Physiologia plantarum.

[22] Wu L (2014). Overexpression of the maize GRF10, an endogenous truncated growth-regulating factor protein, leads to reduction in leaf size and plant height. Journal of integrative plant biology.

[23] Kim JS (2012). Arabidopsis growth-regulating factor7 functions as a transcriptional repressor of abscisic acid- and osmotic stress-responsive genes, including DREB2A. The Plant cell.

[24] Wang L (2011). miR396-targeted AtGRF transcription factors are required for coordination of cell division and differentiation during leaf development in Arabidopsis. Journal of experimental botany.

[25] Liang G, He H, Li Y, Wang F, Yu D (2014). Molecular mechanism of microRNA396 mediating pistil development in Arabidopsis. Plant physiology.

[26] Arvidsson S, Perez-Rodriguez P, Mueller-Roeber B (2011). A growth phenotyping pipeline for *Arabidopsis thaliana* integrating image analysis and rosette area modeling for robust quantification of genotype effects. The New phytologist.

[27] Che R (2015). Control of grain size and rice yield by GL2-mediated brassinosteroid responses. Nature. Plants.

[28] Hu J (2015). A Rare Allele of GS2 Enhances Grain Size and Grain Yield in Rice. Molecular plant.

[29] Franco-Zorrilla JM (2007). Target mimicry provides a new mechanism for regulation of microRNA activity. Nature genetics.

[30] Todesco M, Rubio-Somoza I, Paz-Ares J, Weigel D (2010). A collection of target mimics for comprehensive analysis of microRNA function in *Arabidopsis thaliana*. PLoS genetics.

[31] Claeys H, Inze D (2013). The agony of choice: how plants balance growth and survival under water-limiting conditions. Plant physiology.

[32] Dubois M, Claeys H, Van den Broeck L, Inze D (2017). Time of day determines Arabidopsis transcriptome and growth dynamics under mild drought. Plant Cell Environ.

[33] Clauw P (2015). Leaf responses to mild drought stress in natural variants of Arabidopsis. Plant physiology.

[34] Skirycz A (2011). Survival and growth of Arabidopsis plants given limited water are not equal. Nat Biotechnol.

[35] Cao D (2016). Regulations on growth and development in tomato cotyledon, flower and fruit via destruction of miR396 with short tandem target mimic. Plant Sci.

[36] Duan P (2015). Regulation of OsGRF4 by OsmiR396 controls grain size and yield in rice. Nature. Plants.

[37] Li, S. *et al*. The OsmiR396c-OsGRF4-OsGIF1 regulatory module determines grain size and yield in Rice. Plant biotechnology journal, 10.1111/pbi.12569 (2016). 10.1111/pbi.12569 PMC5095787 27107174

[38] Aguirrezabal L (2006). Plasticity to soil water deficit in *Arabidopsis thaliana*: dissection of leaf development into underlying growth dynamic and cellular variables reveals invisible phenotypes. Plant Cell Environ.

[39] Candaele J (2014). Differential methylation during maize leaf growth targets developmentally regulated genes. Plant physiology.

[40] Bertolini E (2013). Addressing the role of microRNAs in reprogramming leaf growth during drought stress in Brachypodium distachyon. Molecular plant.

[41] Chen L, Wang T, Zhao M, Tian Q, Zhang WH (2012). Identification of aluminum-responsive microRNAs in Medicago truncatula by genome-wide high-throughput sequencing. Planta.

[42] Ding Y, Chen Z, Zhu C (2011). Microarray-based analysis of cadmium-responsive microRNAs in rice (Oryza sativa). Journal of experimental botany.

[43] Ding D (2009). Differential expression of miRNAs in response to salt stress in maize roots. Ann Bot.

[44] Gao P (2010). Over-expression of osa-MIR396c decreases salt and alkali stress tolerance. Planta.

[45] Casadevall R, Rodriguez RE, Debernardi JM, Palatnik JF, Casati P (2013). Repression of growth regulating factors by the microRNA396 inhibits cell proliferation by UV-B radiation in Arabidopsis leaves. The Plant cell.

[46] Fina J (2017). UV-B Inhibits Leaf Growth through Changes in Growth Regulating Factors and Gibberellin Levels. Plant physiology.

[47] Soto-Suarez M, Baldrich P, Weigel D, Rubio-Somoza I, San Segundo B (2017). The Arabidopsis miR396 mediates pathogen-associated molecular pattern-triggered immune responses against fungal pathogens. Scientific reports.

[48] Huot B, Yao J, Montgomery BL, He SY (2014). Growth-defense tradeoffs in plants: a balancing act to optimize fitness. Molecular plant.

[49] Heidel AJ, Clarke JD, Antonovics J, Dong X (2004). Fitness costs of mutations affecting the systemic acquired resistance pathway in *Arabidopsis thaliana*. Genetics.

[50] Schindelin J (2012). Fiji: an open-source platform for biological-image analysis. Nature methods.

[51] Czechowski T, Stitt M, Altmann T, Udvardi MK, Scheible WR (2005). Genome-wide identification and testing of superior reference genes for transcript normalization in Arabidopsis. Plant physiology.

[52] Irwin JA (2016). Nucleotide polymorphism affecting FLC expression underpins heading date variation in horticultural brassicas. Plant J.

[53] Vercruyssen L (2015). GROWTH REGULATING FACTOR5 stimulates Arabidopsis chloroplast division, photosynthesis, and leaf longevity. Plant physiology.

[54] Rizzi, Y. S., Cecchini, N. M., Fabro, G. & Alvarez, M. E. Differential control and function of Arabidopsis ProDH1 and ProDH2 genes on infection with biotrophic and necrotrophic pathogens. *Mol Plant Pathol*, 10.1111/mpp.12470 (2016). 10.1111/mpp.12470 PMC6638284 27526663

[55] Sievers F (2011). Fast, scalable generation of high-quality protein multiple sequence alignments using Clustal Omega. Molecular Systems Biology.

[56] Felsenstein, J. PHYLIP (Phylogeny Inference Package) version 3.6. Distributed by the author. Department of Genome Sciences, University of Washington, Seattle, USA. http://evolution.genetics.washington.edu/phylip.html (2005).

