# Supplementary material for: Robust increase of leaf size by Arabidopsis thaliana GRF3-like transcription factors under different growth conditions
Source: Sci Rep. 2018 Sep 7;8:13447. doi: 10.1038/s41598-018-29859-9 (PMC6128883; doi:10.1038/s41598-018-29859-9)
Supplement: Supplementary file 1 — Supplementary Info [file 41598_2018_29859_MOESM1_ESM.pdf]

Supplementary Information for:

Robust increase of leaf size by *Arabidopsis thaliana GRF3*-like transcription factors under different growth conditions

Matías Beltramino, María Florencia Ercoli, Juan Manuel Debernardi, Camila Goldy, Arantxa M. L. Rojas, Florencia Nota, María Elena Alvarez, Liesbeth Vercruyssen, Dirk Inzé, Javier F. Palatnik1,6, and Ramiro E. Rodriguez.

Supplementary Figure S1. Expression levels of the GRF transcription factors in Arabidopsis thaliana plants expressing rGRF3.

Supplementary Figure S2. Plastochron length in rGRF3 and control plants under mild drought stress.

Supplementary Figure S3. rGRF3 plants are more resistant to Pseudomonas syringae pv. tomato DC3000.

Supplementary Figure S4. Full-length blots used to prepare Figures 2d (Panel a) and 5d (Panel b).

Supplementary Table S1. Distribution of GRFs in selected plants species and the ocurrence of miR396 regulation.

Supplementary Table S2. Selection of soybean and rice GRFs for heterologous expression in Arabidopsis thaliana.

Supplemental Table S3. Relevant Gene Identifiers and oligonucleotide primers used in RT-qPCR.

Supplementary Table S4. Binary plasmids used in this study to generate transgenic lines.

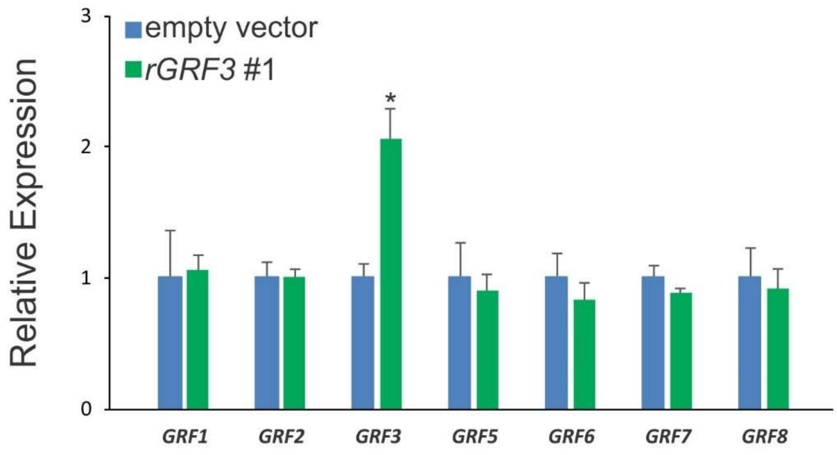

Supplementary Figure S1. Expression levels of the GRF transcription factors in Arabidopsis thaliana plants expressing rGRF3.

Expression levels of the Arabidopsis *GRFs* in homozygous T3 transgenic plants transformed with the empty vector or *rGRF3* (*rGRF3* #1). The gene expression levels were estimated by RT-qPCR and normalized to control plants (empty vector). The data shown are mean ± SEM of three biological replicates. Asterisks indicate significant differences from control plants as determined by Student's t- test ($P < 0.05$).

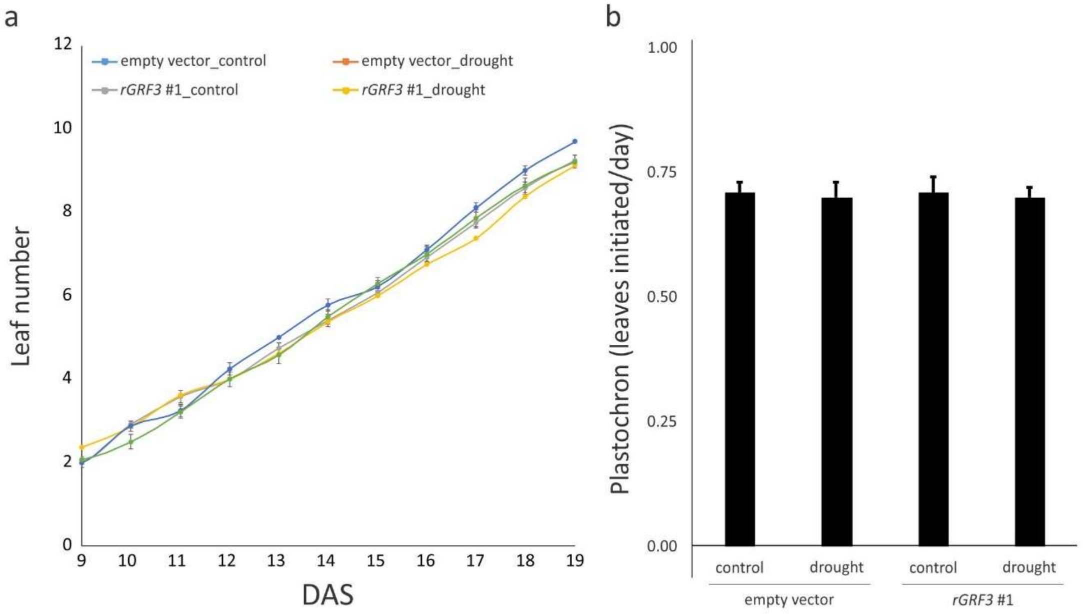

Supplementary Figure S2. Plastochron length in rGRF3 and control plants under mild drought stress.

a. Time course analysys of the number of leaves under well-watered or mild drought conditions of control (empty vector) and *rGRF3* #1 plants.

b. Plastochron length (expressed as the number of leaves initatied per day) in control (empty vector) or *rGRF3* #1 under well-watered or mild drought conditions.

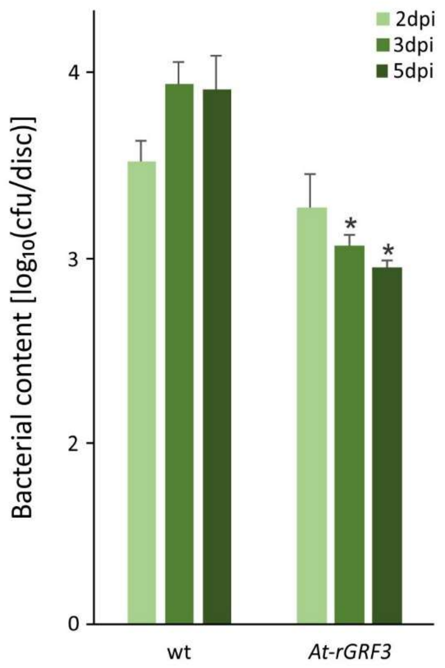

Supplementary Figure S3. rGRF3 plants are more resistant to Pseudomonas syringae pv. tomato DC3000.

Bacterial growth was quantified at 2, 3 and 5 days post infection (dpi) on leaves of adult plants infiltrated with pathogen suspensions (5.10^5 cfu/ml). Similar restults were ontained in 3 biological replicates and the results of a representative experiment is shown. Asterisks indicate significant differences bewteen control and *rGRF3* as determined by Student’s t-test ($P < 0.05$).

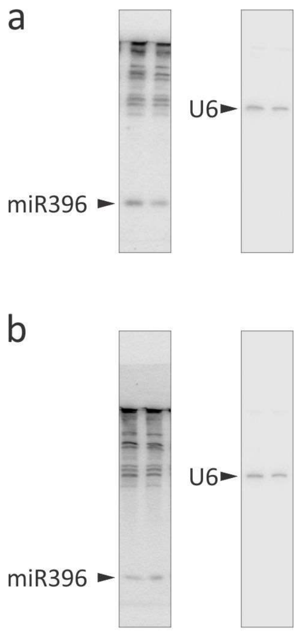

Supplementary Figure S4. Full-length blots used to prepare Figures 2d (Panel a) and 5d (Panel b).

Supplementary Table S1. Distribution of *GRFs* in selected plants species and the ocurrence of miR396 regulation

Especie	Common Name	Number of *GRFs*	miR396 target *GRFs*	% target	non-targets	Data from:	Classification
Glycine max	Soybean	21	21	100	0	PTFDb[a]	Eudicot
Populus trichocarpa	Poplar	19	19	100	0	PTFDb	Eudicot
Brassica rapa	Turnip	17	13	76	4	Wang et al 2014	Eudicot
Manihot esculenta	Cassava	17	15	88	2	PTFDb	Eudicot
Solamum lycopersicum	Tomato	13	12	92	0	PTFDb	Eudicot
Malus domestica	Apple	12	12	100	0	PTFDb	Eudicot
Prunus persica	Peach	10	10	100	0	PTFDb	Eudicot
Theobroma cacao	Cacao	10	10	100	0	PTFDb	Eudicot
Citrus sinensis	Orange	10	10	100	0	PTFDb	Eudicot
Arabidopsis thaliana	Arabidopsis	9	7	78	2	Kim et al 2003	Eudicot
Arabidopsis lyrata	Lyrata	9	7	78	2	PTFDb	Eudicot
Solanum tuberosum	Potato	9	9	100	0	PTFDb	Eudicot
Capsella rubella	Pink shepherd's purse	8	6	75	2	PTFDb	Eudicot
Vitis vinifera	Grape	8	8	100	0	PTFDb	Eudicot
Carica papaya	Papaya	7	7	100	0	PTFDb	Eudicot
Medicago truncatula	Medicago	6	6	100	0	Bazim et al 2013	Eudicot
Zea mays	Maize	14	12	86	2	Zhang et al 2008	Monocot
Oriza sativa	Rice	12	11	92	1	Choi et al 2004	Monocot
Sorgum bicolor	Sorghum	10	9	90	1	PTFDb	Monocot
Setaria italica	Foxtail millet	11	10	91	1	PTFDb	Monocot
Brachypodiym distachyon	Purple false brom	11	9	82	2	PTFDb	Monocot

[a] PTFDb: Plant Transcription Factor Database (http://planttfdb.cbi.pku.edu.cn/)

Supplementary Table S2. Selection of soybean and rice *GRFs* for heterologous expression in *Arabidopsis thaliana*

Organism	Gene	Source	Similarity to *AtGRF3* (%)	Selected *GRF*
Arabidopsis thaliana	*AtGRF1*	Kim et al (2003) The Plant Journal	36.8	
	AtGRF2		32.6	
	AtGRF3		**100.0**	**(+)**
	AtGRF4		74.8	
	AtGRF5		29.8	
	AtGRF6		26.9	
	AtGRF7		35.9	
	AtGRF8		33.1	
	AtGRF9		26.7	
Oryza sativa	*OsGRF1*	Choi et al. (2004) Plant Cell Physiol	32.0	
	OsGRF2		34.8	
	OsGRF3		32.4	
	OsGRF4		**38.2**	**(+)**
	OsGRF5		32.0	
	OsGRF6		33.3	
	OsGRF7		33.0	
	OsGRF8		32.2	
	OsGRF9		37.4	
	OsGRF10		29.7	
	OsGRF11		21.1	
	OsGRF12		23.7	
Glycine max	*Glyma.01G148600*	PTFDb[a]	38.1	
	Glyma.01G234400		36.9	
	Glyma.03G192200		**58.9**	**(+)**
	Glyma.04G230600		31.6	
	Glyma.07G038400		30.2	
	Glyma.09G068700		32.7	
	Glyma.09G212500		37.0	
	Glyma.10G067200		54.5	
	Glyma.11G008500		37.9	
	Glyma.11G110700		36.6	
	Glyma.11G208800		56.1	
	Glyma.12G014700		36.0	
	Glyma.13G109500		31.9	
	Glyma.15G176500		33.3	
	Glyma.16G007600		32.0	
	Glyma.17G050200		33.0	
	Glyma.17G232600		34.2	
	Glyma.17G232700		34.0	
	Glyma.19G192700		60.3	
	Glyma.U028600		34.0	
	Glyma.U028700		34.4	

[a] PTFDb: Plant Transcription Factor Database (http://planttfdb.cbi.pku.edu.cn/)

Supplemental Table S3. Relevant Gene Identifiers and oligonucleotide primers used in RT-qPCR.

Gene	Locus ID	RT-qPCR Forward primer	RT-qPCR Reverse primer
AtGRF1	At2g22840	ATGGAAAGAAATGGCGGTGCTC	CGGCGGCAGCATTAGTATTGT
AtGRF2	At4g37740	CACATCAACAGAGGCCGTCATCG	AACCGGAGATTCCTTGGGTTGTAAG
AtGRF3	At2g36400	GTCTTCGCTGGCCACAAGTATT	TGTTGCTGTTGTAGTGGTGGCT
AtGRF5	At3g13960	CTCTTCATCATGCTTCCGCTTT	TTGCTAACGGTTGTTGGTGATG
AtGRF6	At2g06200	TCCTCAAGAAAGCCTCCTCCTA	ATCTTCCATTGCTGAGCCAGAG
AtGRF7	At5g53660	TGCGCGAAAGAAGTCGTCTCTA	CACCATTGTTGTTAGGGCGAGA
AtGRF8	At4g24150	GCATGTGGAATCATCTCACCAA	TCTCCGGGTTTAAGGTTCCAAT
PP2A	At1g13320	CCTGCGGTAATAACTGCATCT	CTTCACTTAGCTCCACCAAGCA
UBC	-	GATCCACCCACCTCGTGTAG	CTGGAGGGAAGTGAATGGTAAC
OsGRF4	Os02g47280	-	-
GmGRF	Glyma.03g192200	-	-

Supplementary Table S4. Binary plasmids used in this study to generate transgenic lines.

Reporter and protein expression vectors.

Vector	Construct	Arabidopsis Chromosome: start-end[a]
RER32	*rGRF3*<br>*(pGRF3-rGRF3)*	*pGRF3:rGRF3*; 2: 15273644 – 15270078<br>CGC AAC CGT TCT AGA AAA CCA GTA GAG ACT CCA<br>R N R S R K P V E T P
RER35	*rGRF2*<br>*(pGRF2-rGRF2)*	*pGRF2:rGRF2*; 4: 17729690 - 17725303<br>CGT CAT CGT TCT AGA AAA CCG GTC GAA GTC CAA<br>R H R S R K P V E V Q
RER149	*pGRF3-rGRF2*	*pGRF3* (2: 15273644 - 15272819) – *rGRF2* (4: 17727612 - 17725536)
RER160	*At-rGRF3*	*pGRF3* (2: 15273644 - 15272819) – *At-rGRF3* CDS[b]
RER159	*Gm-rGRF*	*pGRF3* (2: 15273644 - 15272819) – *Gm-rGRF* CDS[c]
RER158	*Os-rGRF4*	*pGRF3* (2: 15273644 - 15272819) – *Os-rGRF4* CDS[d]

miR396 and *MIM396* expression vectors.

Vector	Construct	Expressed sequence[e]
JP123	*35S:miR396b*	GGATCCCCCTAGAGTGGTCCTCTTGAGTTTCTTCTATATGCTAGTTGCTCAACTAATATCT<br>CTCTCTCTATCTCTCAACAATTCTCTTAAGTTTAATTAGTTTTCAGAAGAAGGAGAAGATG<br>AAGATCCTGGTCATACTTTTCCACAGCTTTCTTGAACTTTCTTTTTCATTTCCATTGTTTT<br>TTTCTTAAACAAAAGTAAGAAGAAAAAAAACTTTAAGATTAAGCATTTTGGAAGCTCAAGA<br>AAGCTGTGGGAAAACATGACAATTCAGGGTTTACTCCATTGATTCACTTGTCAAGATTCT<br>TCTTTCCAAAGATATTGATTTGAAGGTATGGTTACATAAACCCTTTATTCCTAAATTGAAG<br>ACACAGGATAACTCTTTAGATCCATAATTATGGTCAGAATTATGGATCTAAAGAGTCTGCA
MB11	*35S:9x-MIM396*	GATCCGCCGTAGCCGGCAGGTCTTCTCCCTCTAGAAATTGTTCAAGAGCTCAGCTGTGGAA<br>AGCTTCGGTTTTTCTCTTTGGAATGTTCAAGAGCGCAGCTGTGGAATTTTTCAATTTTTTT<br>GGTTGGAATGTTCAAGAGCTTAGCTGTGGAATTTTGATGGAAGATCCGCCGTAGCCGGCAG<br>GTCTTCTCCCTCTAGAAATTGTTCAAGAGCTCAGCTGTGGAAAGCTTCGGTTTTTCTCTTT<br>GGAATGTTCAAGAGCGCAGCTGTGGAATTTTTCAATTTTTTTGGTTGGAATGTTCAAGAGC<br>TTAGCTGTGGAATTTTGATGGAAGATCCGCCGTAGCCGGCAGGTCTTCTCCCTCTAGAAAT<br>TGTTCAAGAGCTCAGCTGTGGAAAGCTTCGGTTTTTCTCTTTGGAATGTTCAAGAGCGCAG<br>CTGTGGAATTTTTCAATTTTTTTGGTTGGAATGTTCAAGAGCTTAGCTGTGGAATTTTGAT<br>GGAAGATC

All constructs were cloned in the binary vector pCHF3 (Jarvis, P., Chen, L. J., Li, H., Peto, C. A., Fankhauser, C., and Chory, J. (1998). An Arabidopsis mutant defective in the plastid general protein import apparatus. Science 282, 100103). T-DNA constructs were introduced into *A. tumefaciens* strain ASE (Fraley, R. T., Rogers, S. G., Horsch, R. B., Eichholtz, D. A., Flick, J. S., Fink, C. L., Hoffmann, N. L., and Sanders, P. R. (1985) The SEV system: a new disarmed Ti plasmid vector system for plant transformation. Biotechnology 3, 629635).

[a] Highlighted in yellow, nucleotides annealing with miR396. Underlined, mutagenized residues. In red, upstream and downstream sequences.

[b] *At-rGRF3* : ATGGATTTGCAACTGAAACAATGGAGAAGCCAGCAGCAGCAACAACATCAGACAGAGTCAGAAGAACAACCTTCTGCAG
CTAAGATACCAAAACATGTCTTTGACCAGATTCATTCTCACACTGCAACTTCTACTGCTCTTCCTCTCTTTACCCCTGAGCCTACTTCTTCT
AAACTCTCCTCTTTGTCTCCTGATTCTTCCTCCAGGTTCCCCAAGATGGGGAGCTTCTTTAGCTGGGCACAGTGGCAAGAACTTGAACTA
CAAGCTCTGATCTACAGGTACATGTTGGCTGGTGCTGCTGTTCCTCAGGAGCTCCTTTTACCAATCAAGAAAAGCCTTCTCCATCTATCTC
CTTCCTACTTTCTTCACCATCCTCTTCAACACCTACCTCATTACCAACCTGCTTGGTATTTGGGAAGGGCAGCGATGGATCCTGAGCCAGG
CAGATGCAGGAGAACGGATGGTAAGAAGTGGAGATGTTCAAGAGACGTCTTCGCTGGCCACAAGTATTGCGAGCGCCACATGCACCG
TGGCCGCAACCGTTCtAGAAAaCCaGTaGAgACTCCAACCACCGTCAATGCAACTGCCACGTCCATGGCTTCATCAGTAGCAGCCGCAGC
CACCACTACAACAGCAACAACAACATCTACGTTTGCTTTTGGTGGTGGTGGTGGTAGTGAGGAAGTGGTTGGTCAAGGAGGATCTTTCT
TCTTCTCTGGCTCTTCTAACTCTTCATCTGAACTTCTCCACCTTAGTCAAAGTTGTTCGGAGATGAAGCAAGAAAGCAACAACATGAACA
ACAAGAGGCCATACGAGTCCCACATCGGATTCAGTAACAACAGATCAGATGGAGGACACATCCTGAGGCCCTTCTTTGACGATTGGCCT
CGTTCTTCGCTCCAAGAAGCTGACAATAGTTCAAGCCCCATGAGCTCAGCCACTTGTCTCTCCATCTCCATGCCCGGGAACTCTTCCTCA
GACGTCTCTCTGAAGCTGTCCACAGGCAACGAAGAGGGAGCCCGGAGCAACAACAATGGGAGAGATCAGCAAAACATGAGCTGGTG
GAGCGGTGGAGGTTCCAACCACCATCATCACAACATGGGCGGACCATTGGCCGAAGCCCTGAGATCTTCTTCCTCATCTTCCCCAACCA
GTGTTCTCCATCAGCTTGGTGTCTCGACACAAGCCTTTCATTGA

[c] *Gm-rGRF*: ATGGACTTCCATCTGAAGCAATGGAGAAACCAGCACGAGTCAGAGGAACAACATTCTACAAAGATGCCAAAACTTCTCCC
TGAATCCCATCAACAACAACAGCCATCAGCCTCTGCACTCCCTTTGTTTGTACCTGAACCCAACAGCAGCAAAGTCAGCACCCTATTATTT
CCCAGGATGGGGAGCTACTTCAGCTTGTCTCAGTGGCAGGAGCTTGAGTTGCAGGCTTTGATATTCAGGTACATGTTGGCTGGTGCTGC
TGTTCCTCCTGAACTCCTTCAACCAATCAAGAAAAGCCTTCTTCATTCTCCACACTATTACCTCCATCACCCTCTCCAACATTACCAACCTTC
TGCTTGGTATTGGGGTAGAGGAGCGATGGATCCGGAGCCAGGGCGGTGCCGGAGAACCGACGGCAAGAAGTGGCGCTGTTCGAGGG
ACGTGGTGGCTGGGCAAAAGTACTGTGAGCGCCACATGCACCGTGGAAGAAACCGTTCtAGAAAaCCaGTaGAgCTACCCACACCAACT
AGTGCTATTAACAATTGTGGTGTAACTGGAGTTGGATCCCTAGGACCAGGTGCTTCATCATCTTCCATTTGTTCACCACCCTTAGCTTCTG
CTTCATTCAAATCTCCTTTTGATCTTCATCTTGATGAACGTTCCTCTGGGACCAAGAATGAAGACGAAGATCATGTGGGTGGGGATGGC
AGATCAGGTGGAGGTGGTGGCCATATGCTGAGGCATTTCTTCGATGATTGGCCACGATCACTCCAAGACTCTGACAACGTTGAAAACA
ATGCTGCTGCTGGCCGTAGCCTCTCTATTTCAATGCCCGGTGCTTCCTCGGATGTGTCATTGAAATTGTCCACGGGCTATGGAGAGGACT
CGGGCCCAGGAAATGAGAATGTAAGCCTCGAGCCAGAGCAGCTGCAGTTGAATTGGGCCGGAGGATGGGCCTCGTCTAATCAAGTGG
CTTCGATGGGAGGTCCACTTGCTGAGGCACTCAGATCATCTACTTCAACCTCATCTCCCACTAGTGTTTTGCATCGTCACTTGCCTCGTGG
ATCTGAGACCAGCTTTATTAGCACCTGA

[d] *Os-rGRF4*: ATGCCTCCCTGTCTCCGGCGGTGGCCGACCACCGCTCGTCCCCGGCAGCCGCGACCGCCTCCCTCCTCCCCTTCTGCCGCT
CCACCCCGCTCTCCGCGTAAGCAACGCGAACCCGCGGCTACAACCCATTTTCTTGGCTCCAGTGGTGCATGTGACAACACGGTGAGACG
TTGTGTGTGGGTGGGTGGGTGCAGGGGCGGTGGTGGCGTCGCGATGGGGGAGGACGCGCCGATGACCGCGAGGTGGCCGCCGGCG
GCGGCGGCGAGGCTGCCGCCGTTCACCGCGGCGCAGTACGAGGAGCTGGAGCAGCAGGCGCTCATATACAAGTACCTGGTGGCAGG
CGTGCCCGTCCCGCCGGATCTCGTGCTCCCCATCCGCCGCGGACTCGACTCCCTCGCCGCCCGCTTCTACAACCATCCCGCCCTTGGATA
TGGTCCGTACTTCGGCAAGAAGCTGGACCCAGAGCCAGGGCGGTGCCGGCGTACGGACGGCAAGAAATGGCGGTGCTCGAAGGAGG
CCGCGCCGGATTCCAAGTACTGCGAGCGCCACATGCACCGCGGCCGCAACCGTTCtAGAAAaCCaGTaGAgACGCAGCTGGTCGCCCAG
TCCCAACCGCCCTCATCTGTTGTCGGTTCTGCGGCGGCGCCCCTTGCTGCTGCCTCCAATGGCAGCAGCTTCCAAAACCACTCTCTTTACC
CTGCTATTGCCGGCAGCAATGGCGGGGGCGGGGGGAGGAACATGCCCAGCTCATTTGGCTCGGCGTTGGGTTCTCAGCTGCACATGG
ATAATGCTGCCCCTTATGCAGCTGTTGGTGGTGGAACAGGCAAAGATCTCAGGTATACTGCTTATGGCACAAGATCTTTGGCGGATGA
GCAGAGTCAACTCATTACTGAAGCTATCAACACATCTATTGAAAATCCATGGCGGCTGCTGCCATCTCAGAACTCGCCATTTCCCCTTTC
AAGCTATTCTCAGCTGGGGGCACTAAGTGACCTTGGTCAGAACACCCCCAGCTCACTTTCAAAGGTTCAGAGGCAGCCACTTTCGTTCTT
TGGGAACGACTATGCGGCTGTCGATTCTGTGAAGCAAGAGAACCAGACGCTGCGTCCCTTCTTTGATGAGTGGCCAAAGGGAAGGGA
TTCATGGTCAGACCTCGCTGATGAGAATGCTAATCTTTCGTCATTCTCAGGCACCCAACTGTCGATCTCCATACCAATGGCATCCTCTGAC
TTCTCGGCGGCCAGTTCTCGATCAACTAATGGTGACTGA

[e] Highlighted in yellow, the mature miRNA sequence expressed. In red, mutagenized residues. Underlined, the target mimicry sequence against miR396.
